# Recombinant fusion protein of cholera toxin B subunit with YVAD secreted by *Lactobacillus casei* inhibits lipopolysaccharide-induced caspase-1 activation and subsequent IL-1 beta secretion in Caco-2 cells

**DOI:** 10.1186/1472-6750-14-38

**Published:** 2014-05-10

**Authors:** Yukihiro Hiramatsu, Masatatsu Yamamoto, Tomomitsu Satho, Keiichi Irie, Akiko Kai, Saori Uyeda, Yuki Fukumitsu, Akihisa Toda, Takeshi Miyata, Fumio Miake, Takeshi Arakawa, Nobuhiro Kashige

**Affiliations:** 1 Faculty of Pharmaceutical Sciences, Fukuoka University, 8-19-1, Nanakuma, Jonan-ku, Fukuoka, Japan; 2 Department of Molecular Oncology, Graduate School of Medical and Dental Sciences, Kagoshima University, 8-35-1, Sakuragaoka, Kagoshima, Japan; 3 Daiichi College of Pharmaceutical Sciences, 22-1, Tamagawa-cho, Minami-ku, Fukuoka, Japan; 4 Division of Molecular Functions of Food, Department of Biochemistry and Biotechnology, Kagoshima University, 1-21-24, Korimoto, Kagoshima, Japan; 5 Molecular Microbiology Group, Department of Tropical Infectious Diseases, COMB, Tropical Biosphere Research Center, University of the Ryukyus, 1 Senbaru, Nishihara, Okinawa, Japan; 6 Division of Host Defense and Vaccinology, Department of Microbiology, Graduate School of Medicine, University of the Ryukyus, 207 Uehara, Nishihara, Okinawa, Japan

**Keywords:** Caspase-1, Cholera toxin B subunit, GM1 ganglioside, Interleukin-1β, *Lactobacillus casei*, YVAD

## Abstract

**Background:**

*Lactobacillus* species are used as bacterial vectors to deliver functional peptides to the intestine because they are delivered live to the intestine, colonize the mucosal surface, and continue to produce the desired protein. Previously, we generated a recombinant *Lactobacillus casei* secreting the cholera toxin B subunit (CTB), which can translocate into intestinal epithelial cells (IECs) through GM1 ganglioside. Recombinant fusion proteins of CTB with functional peptides have been used as carriers for the delivery of these peptides to IECs because of the high cell permeation capacity of recombinant CTB (rCTB). However, there have been no reports of rCTB fused with peptides expressed or secreted by *Lactobacillus* species. In this study, we constructed *L. casei* secreting a recombinant fusion protein of CTB with YVAD (rCTB–YVAD). YVAD is a tetrapeptide (tyrosine–valine–alanine–aspartic acid) that specifically inhibits caspase-1, which catalyzes the production of interleukin (IL)-1β, an inflammatory cytokine, from its inactive precursor. Here, we examined whether rCTB–YVAD secreted by *L. casei* binds to GM1 ganglioside and inhibits caspase-1 activation in Caco-2 cells used as a model of IECs.

**Results:**

We constructed the rCTB–YVAD secretion vector pSCTB–YVAD by modifying the rCTB secretion vector pSCTB. *L. casei* secreting rCTB–YVAD was generated by transformation with pSCTB–YVAD. Both the culture supernatant of pSCTB–YVAD-transformed *L. casei* and purified rCTB–YVAD bound to GM1 ganglioside, as did the culture supernatant of pSCTB-transformed *L. casei* and purified rCTB. Interestingly, although both purified rCTB–YVAD and rCTB translocated into Caco-2 cells, regardless of lipopolysaccharide (LPS), only purified rCTB–YVAD but not rCTB inhibited LPS-induced caspase-1 activation and subsequent IL-1β secretion in Caco-2 cells, without affecting cell viability.

**Conclusions:**

The rCTB protein fused to a functional peptide secreted by *L. casei* can bind to GM1 ganglioside, like rCTB, and recombinant YVAD secreted by *L. casei* may exert anti-inflammatory effects in the intestine. Therefore, rCTB secreted by *L. casei* has potential utility as a vector for the delivery of YVAD to IECs.

## Background

Lactic acid bacteria (LAB) are not pathogenic and their classification is “generally recognized as safe”. Over the past several decades, LAB have been used in foods and medicines because they confer beneficial effects on the health of the host. Moreover, after their administration, LAB are delivered live to the intestine, colonizing the mucosal surface and exerting various effects [1]. Therefore, LAB that produce heterologous proteins have been used as bacterial vectors for the delivery of functional proteins to the intestine. Many studies using recombinant DNA technology have used *Lactobacillus* species, which are present in large numbers in the human gut and are resistant to gastric and bile acids [2]. These live recombinant lactobacilli colonize the intestinal mucosal surface and produce the desired protein [3]. Although *Escherichia coli* has generally been used for the production of heterologous proteins, coliform lipopolysaccharide (LPS) contamination always poses a problem. In contrast to *E. coli*, *Lactobacillus* species are gram-positive bacteria and consequently do not contain LPS. Therefore, we selected *Lactobacillus* species for the secretion of functional heterologous proteins.

Cholera toxin (CT) is an enterotoxin produced by *Vibrio cholerae*, which is composed of a toxic A subunit (CTA) and nontoxic B subunit (CTB). CT gains entry to intestinal epithelial cells (IECs) when CTB binds to GM1 ganglioside, a cell-surface receptor present on mammalian cells. CTB alone can translocate into IECs through the GM1 ganglioside without toxicity [4]. Many groups have reported that recombinant CTB (rCTB) expressed in various bacteria, yeasts, and plants also binds to GM1 ganglioside. Previously, we constructed a recombinant *Lactobacillus casei* that secretes CTB, and showed that the rCTB secreted by *L. casei* has GM1-ganglioside-binding activity similar to that of CT from *V. cholerae*[5]. Recombinant fusion proteins of CTB with functional proteins and peptides, such as vaccine antigens [6] and the insulin B chain peptide [7], have been used as carriers to deliver these proteins and peptides to IECs, because they also bind to GM1 ganglioside. However, it has not been determined whether recombinant fusion proteins of CTB with functional proteins or peptides expressed by *Lactobacillus* species bind GM1 ganglioside and translocate into IECs.

The synthetic tetrapeptide composed of tyrosine, valine, alanine, and aspartic acid (YVAD) is a specific inhibitor of caspase-1 [8]. Caspase-1 catalyzes the production of interleukin (IL)-1β, an inflammatory cytokine, from its precursor (pro-IL-1β), and its overexpression in and secretion from IECs exacerbates intestinal inflammation [9,10]. Caspase-1 is also produced as an inactive precursor, pro-caspase-1, which is activated by inflammatory stimuli, such as LPS and mature caspase-1 itself [11,12]. Therefore, YVAD has anti-inflammatory properties, acting as a decoy substrate for caspase-1 instead of pro-IL-1β and pro-caspase-1. However, recombinant bacteria expressing or secreting YVAD have not been reported because it is difficult to express and secrete recombinant low-molecular-weight peptides in bacteria. Furthermore, for YVAD to inhibit caspase-1 activation and subsequent IL-1β secretion, it must be translocated into IECs. However, the cell permeation capacity of YVAD is low because of its strong polarity [13]. Here, we investigated whether fusing rCTB to YVAD would allow the secretion of recombinant YVAD from *L. casei* and facilitate the translocation of YVAD into IECs.

In this study, we constructed *L. casei* that secretes a recombinant fusion protein of CTB with YVAD (rCTB–YVAD) and confirmed that rCTB–YVAD secreted by *L. casei* binds to GM1 ganglioside, translocates into human epithelial colorectal adenocarcinoma Caco-2 cells used as a model of IECs, and inhibits the activation of caspase-1 and subsequent IL-1β secretion from Caco-2 cells.

## Results and discussion

### Secretion of rCTB-YVAD by *L. casei* transformed with pSCTB-YVAD

**Figure 1:**
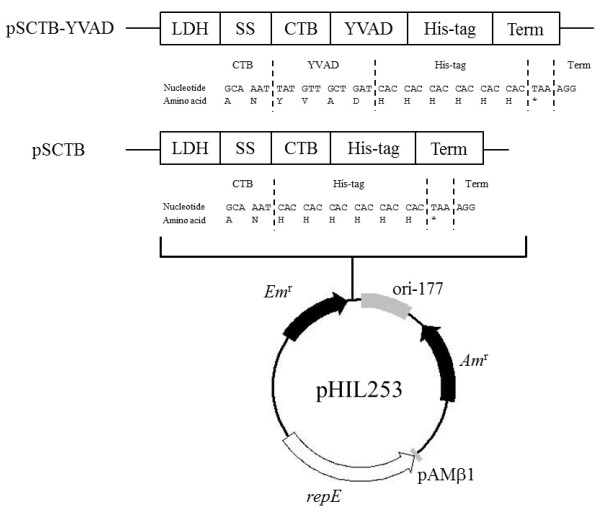
**Construction of rCTB–YVAD secretion vector pSCTB–YVAD.** pSCTB–YVAD includes the promoter of the *LDH* gene, SS of the β-*N*-acetylglucosaminidase gene, the CTB–YVAD-coding sequence, His-tag-coding sequence, and the Term of the β-*N*-acetylglucosaminidase gene in the *Lactobacillus*–*E. coli* shuttle vector pHIL253. The nucleotide and amino acid sequences around the CTB/YVAD fusion site are shown.

**Figure 2:**
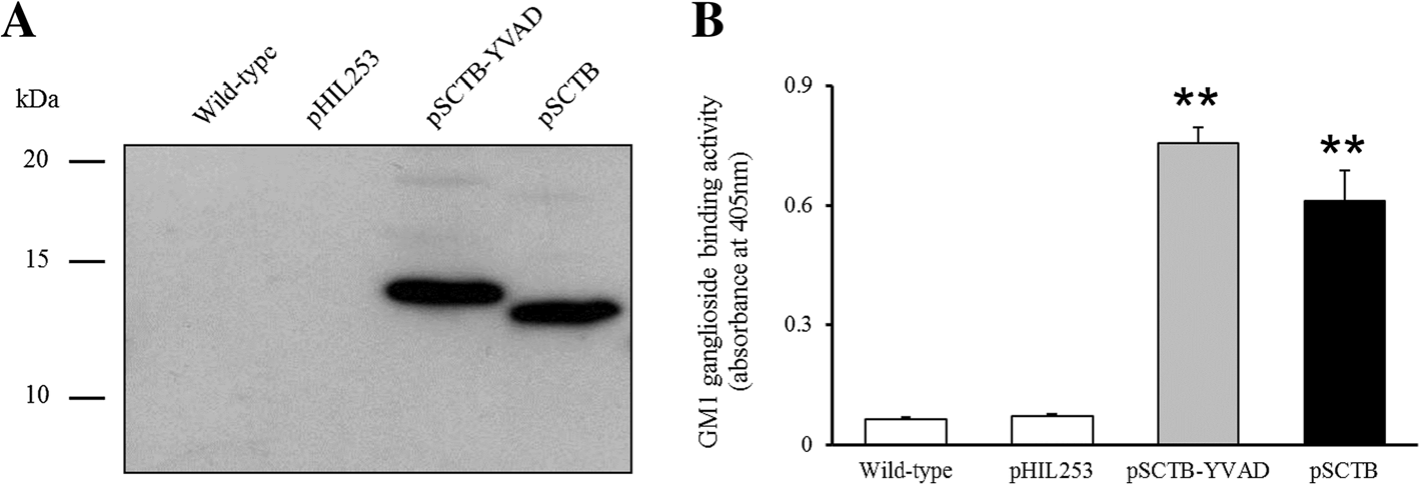
**Secretion and GM1-ganglioside-binding activity of rCTB–YVAD.** Wild-type *L. casei* and *L. casei* transformed with pHIL253, pSCTB–YVAD, or pSCTB were grown at 30°C. The culture supernatants were collected and concentrated 10-fold. Immunoblotting with an antibody directed against CT was performed to analyze the secretion of rCTB–YVAD or rCTB into the supernatant. Data are representative of three separate experiments **(A)**. The GM1-ganglioside-binding activity in the supernatant was analyzed with GM1-ELISA. Data represent the means ± SEM of three separate experiments performed in triplicate **(B)**. **P < 0.01 compared with pHIL253.

Recombinant fusion proteins of CTB with functional peptides expressed in various bacteria [7,14], yeasts [6], and plants [15] have been reported to bind GM1 ganglioside. However, there have been no reports of recombinant fusion proteins of CTB with functional peptides expressed in *Lactobacillus* species. Liljegvist *et al*. reported that the fusion of the serum albumin binding region (BB, approximately 25 kDa) to the C-terminus of CTB had no effect on the GM1-ganglioside-binding activity of CTB, whereas this activity was abolished when BB was fused to the N-terminus of CTB [16]. These observations demonstrate the importance of fusing the functional peptide to the C-terminus of CTB to retain its GM1-ganglioside-binding activity. In contrast, Dertzbaugh and Cox reported that CTB can bind to nickel ions without a His-tag because of a specific histidine residue within its sequence [17]. However, we recently showed that the purification of rCTB with a His-tag from the culture supernatant of *L. casei* was 20 times more efficient than the purification of rCTB without a His-tag [5]. The GM1-ganglioside-binding activity of rCTB with a His-tag, secreted by *L. casei*, was similar to those of rCTB without a His-tag and native CT from *V. cholerae*[5]. Therefore, to avoid any steric effects on the structure and GM1-ganglioside-binding activity of rCTB secreted by *L. casei*, we fused YVAD and the His-tag to the C-terminus of CTB. The rCTB–YVAD secretion vector pSCTB–YVAD includes the promoter region of the lactate dehydrogenase (LDH) gene, the secretory signal sequence (SS) and terminator region (Term) of the β-*N*-acetylglucosaminidase gene from *L. casei*, the CTB-coding sequence from *V. cholerae*, the YVAD-coding sequence (tatgttgctgat; this nucleotide sequence was determined by reference to the codon usage of *L. casei* ATCC 334 and ATCC 393), and the His-tag-coding sequence in the *Lactobacillus*–*E. coli* shuttle vector pHIL253 [5] (Figure 1). Protein bands of about 12–14 kDa were detected in the supernatant of *L. casei* transformed with pSCTB–YVAD or pSCTB, when immunoblotted with an antibody directed against CT (Figure 2A). These results indicate that rCTB–YVAD and rCTB, with predicted molecular weights of 13,231 and 12,783, respectively, were secreted by *L. casei* transformed with pSCTB–YVAD and pSCTB, respectively. Furthermore, the supernatant of *L. casei* transformed with pSCTB–YVAD showed strong binding activity to GM1 ganglioside compared with that of pHIL253 (0.78  ±  0.04 *vs* 0.06  ±  0.01 at OD_405_, respectively, *P*  <  0.01; Figure 2B). This GM1-ganglioside-binding activity was similar to that of the supernatant of *L. casei* transformed with pSCTB (0.61  ±  0.08 at OD_405_). These observations indicate that rCTB–YVAD secreted by *L. casei* transformed with pSCTB–YVAD has binding activity for GM1 ganglioside similar to that of rCTB secreted by *L. casei* transformed with pSCTB.

### Purification of rCTB–YVAD secreted by pSCTB–YVAD-transformed *L. casei*

**Figure 3:**
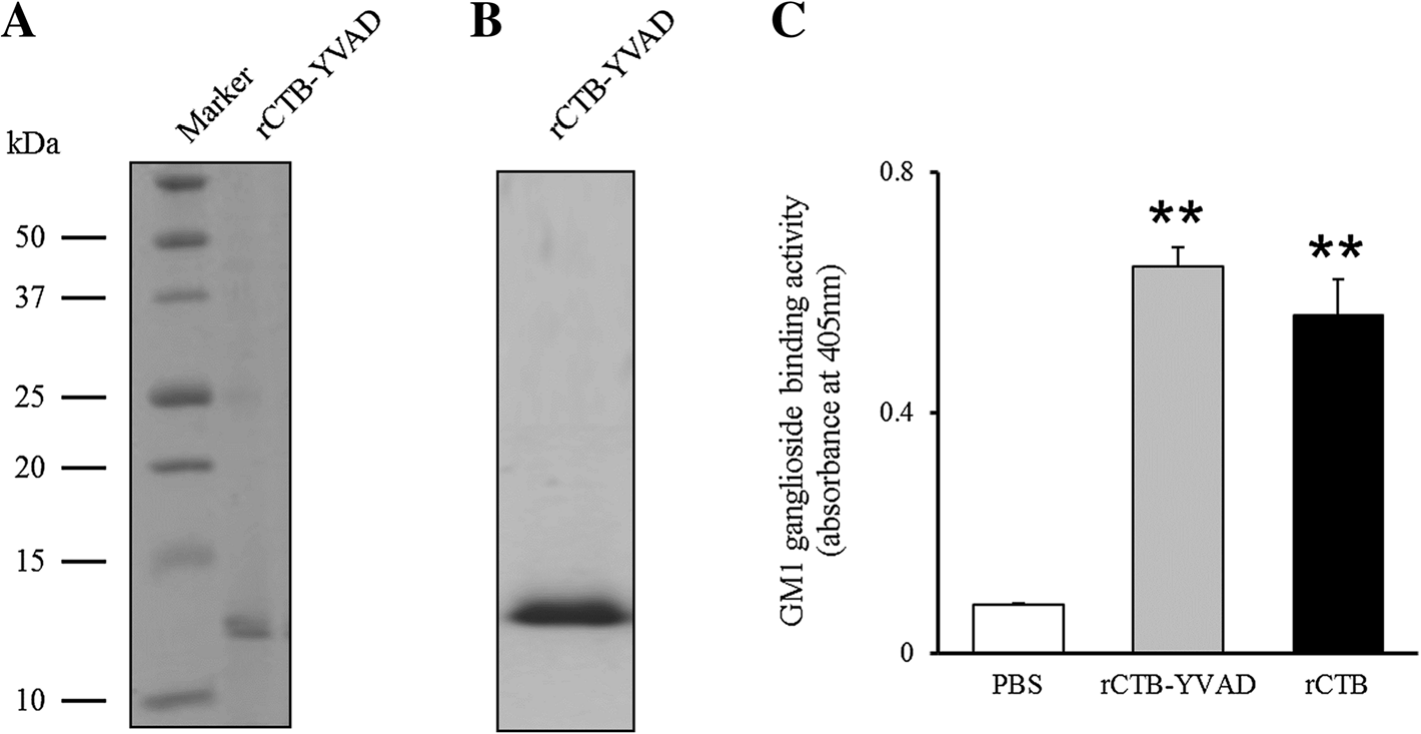
**Purification of rCTB–YVAD from the culture supernatant of *L. casei *transformed with pSCTB–YVAD.** rCTB–YVAD was purified using its His-tag with a nickel-bound affinity resin. Purification was confirmed by SDS-PAGE followed by CBB staining **(A)** and immunoblotting with an antibody directed against CT **(B)**. Data are representative of three separate experiments. The GM1-ganglioside-binding activities of purified rCTB–YVAD and rCTB were analyzed by GM1-ELISA. Data represent the means ± SEM of three separate experiments performed in triplicate **(C)**. **P < 0.01 compared with PBS.

To analyze the inhibitory effect of rCTB–YVAD secreted by *L. casei* transformed with pSCTB–YVAD, it was necessary to coincubate Caco-2 cells with *L. casei* secreting rCTB–YVAD. However, wild-type *L. casei* and *L. casei* transformed with pHIL253, pSCTB–YVAD, or pSCTB do not grow in minimum essential medium (MEM), which is used to culture Caco-2 cells, but grow in MRS/K (see Additional file 1: Figure S1A). Furthermore, neither rCTB–YVAD nor rCTB was detected in the supernatant of *L. casei* transformed with pSCTB–YVAD or pSCTB, respectively, when grown in MEM, but both were detected when the transformants were grown in MRS/K (see Additional file 1: Figure S1B). Therefore, to analyze the inhibitory effect of rCTB–YVAD on LPS-induced caspase-1 activation and subsequent IL-1β secretion, we purified rCTB–YVAD from the culture supernatant of *L. casei* transformed with pSCTB–YVAD using its His-tag and a nickel-bound affinity resin. The purified rCTB–YVAD migrated as a single band on SDS-PAGE when visualized with Coomassie Brilliant Blue (CBB) staining (Figure 3A). Furthermore, purified rCTB–YVAD was detected by immunoblotting with an antibody directed against CT (Figure 3B) and bound more strongly to GM1 ganglioside than did PBS (0.64 ± 0.03 *vs* 0.08 ± 0.01 at OD_405_, respectively, *P* < 0.01; Figure 3C), similar to the supernatant of *L. casei* transformed with pSCTB–YVAD (Figure 2B). The GM1-ganglioside-binding activity of rCTB–YVAD was similar to that of rCTB (0.56 ± 0.06 at OD_405_; Figure 3C). Purified rCTB–YVAD and rCTB were used in further experiments. About 1 mg of rCTB–YVAD protein was obtained from one liter of culture supernatant from *L. casei* transformed with pSCTB–YVAD. This purification efficiency was similar to that for rCTB purified from the culture supernatant of pSCTB-transformed *L. casei*.

### Viability of Caco-2 cells after rCTB–YVAD and rCTB treatment

**Figure 4:**
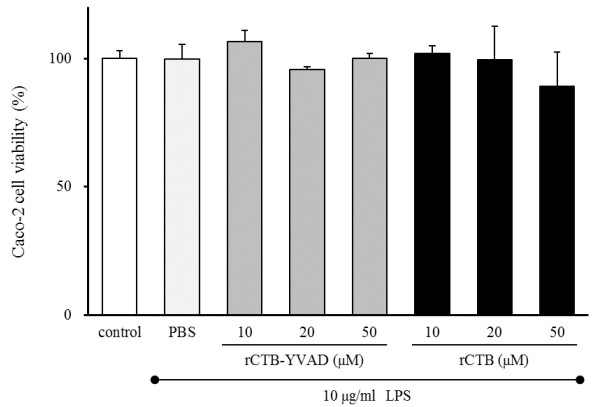
**Viability of Caco-2 cells treated with rCTB–YVAD or rCTB.** Caco-2 cells were treated with three concentrations of rCTB–YVAD or rCTB (10, 20, or 50 μM) for 48 h in the presence of 10 μg/ml LPS. Caco-2 cell viability was measured with the WST-1 method and by comparing the absorbance of untreated control cells with that of PBS-, rCTB–YVAD-, and rCTB-treated cells. Data represent the means ± SEM of three separate experiments performed in triplicate.

There were no significant differences in the viability of Caco-2 cells treated with PBS, rCTB-YVAD, or rCTB in the presence of LPS (Figure 4). Therefore, rCTB–YVAD and rCTB had no effect on Caco-2 cell viability in the presence of LPS. Consequently, rCTB–YVAD and rCTB were used at concentrations of 50 μM in subsequent experiments.

### Translocation of rCTB-YVAD and rCTB into Caco-2 cells

**Figure 5:**
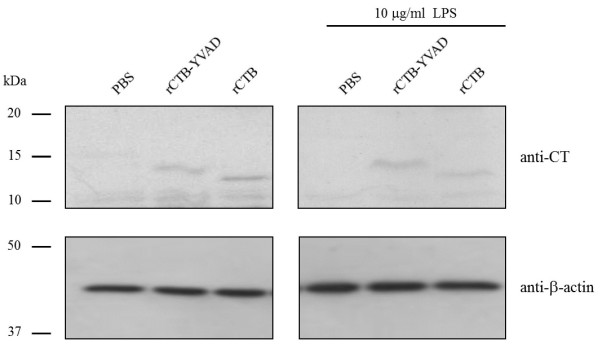
**Translocation of rCTB–YVAD or rCTB into Caco-2 cells.** Caco-2 cells were treated with 50 μM rCTB–YVAD or rCTB for 6 h in the absence or presence of 10 μg/ml LPS. The translocation of rCTB–YVAD or rCTB into Caco-2 cells was detected with immunoblotting using an antibody directed against CT. β-actin was used as the internal control. Data are representative of three separate experiments.

The translocation of rCTB–YVAD is a prerequisite for the inhibition of LPS-induced caspase-1 activation and subsequent IL-1β secretion. Therefore, we examined whether rCTB–YVAD translocates into Caco-2 cells. rCTB–YVAD was detected within Caco-2 cells in the absence or presence of LPS (Figure 5). Similarly, rCTB was detected within Caco-2 cells. These results indicate that both rCTB–YVAD and rCTB are translocated into Caco-2 cells, regardless of LPS treatment.

The cell permeation of functional peptides, such as vaccine antigens and the insulin B chain peptide, is increased by their fusion with CTB [6,7], because CTB translocates easily into IECs by binding to GM1 ganglioside [4]. For YVAD to inhibit caspase-1 activation and subsequent IL-1β secretion, it is necessary for YVAD to translocate into Caco-2 cells. Our results suggest that the fusion of CTB to YVAD contributed to the translocation of YVAD into Caco-2 cells. The translocation of rCTB–YVAD into the cells, regardless of the presence or absence of LPS, suggests that rCTB–YVAD translocates into Caco-2 cells through GM1 ganglioside, which is constantly expressed, regardless of the inflammatory status.

### Inhibitory effect of rCTB–YVAD on LPS-induced caspase-1 activation and subsequent IL-1β secretion in Caco-2 cells

**Figure 6:**
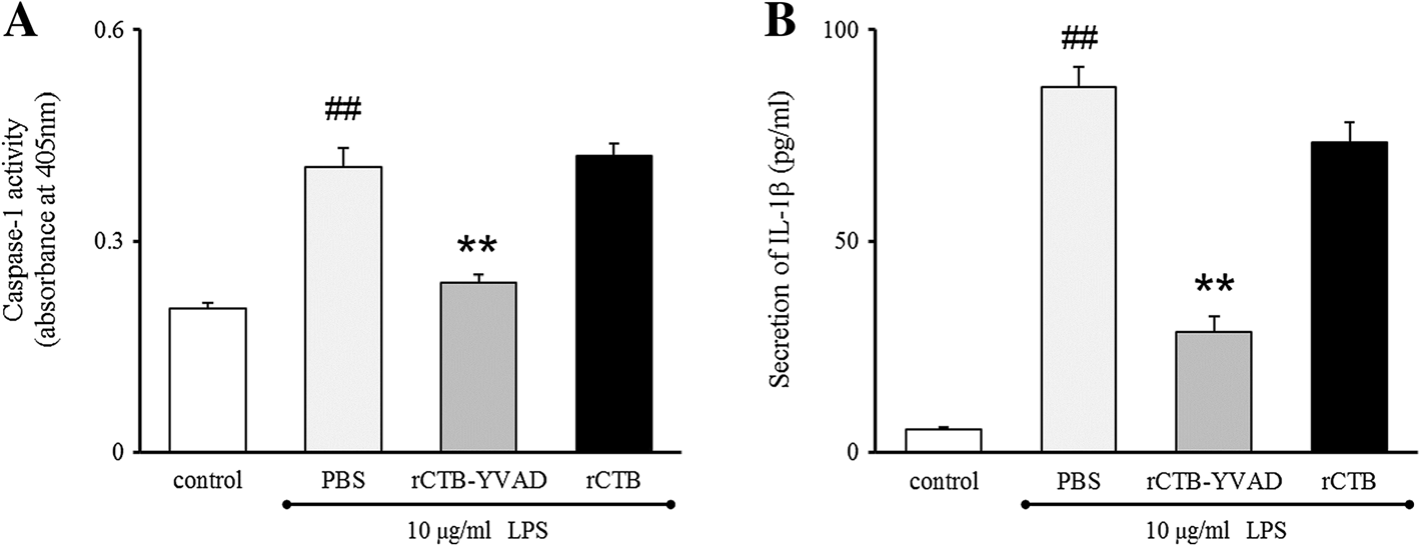
**Inhibitory effects of rCTB–YVAD on LPS-induced caspase-1 activation and IL-1β secretion in Caco-2 cells.** Caco-2 cells were treated with 10 μg/ml LPS for 12 h. The cell lysates were incubated at 30°C for 2 h in the absence or presence of 50 μM rCTB-YVAD or rCTB. Caspase-1 activity was determined with a colorimetric assay **(A)**. Caco-2 cells were treated with 50 μM rCTB–YVAD or rCTB in the presence of 10 μg/ml LPS. The concentrations of IL-1β in the supernatant were determined with an ELISA **(B)**. Data are the means ± SEM of three separate experiments performed in triplicate. ^##^*P* < 0.01 compared with the control, **P < 0.01 compared with LPS  +  PBS treatment.

The level of caspase-1 activity was significantly increased in the lysate of LPS-treated Caco-2 cells compared with that in the untreated control cells (*P* < 0.01). rCTB–YVAD significantly inhibited LPS-induced caspase-1 activation compared with that in cells treated with LPS + PBS (0.24 ± 0.01 *vs* 0.41 ± 0.03 at OD_405_, respectively, *P* < 0.01; Figure 6A). This observation suggests that rCTB-YVAD acts as a decoy substrate for caspase-1. The level of IL-1β secretion increased in Caco-2 cells after LPS treatment compared with that in the untreated controls (*P* < 0.01). Treatment with rCTB–YVAD significantly reduced LPS-induced IL-1β secretion compared with that in cells treated with LPS + PBS (28.5 ± 3.6 *vs* 86.5 ± 4.7 pg/ml, respectively, *P* < 0.01; Figure 6B). In contrast, treatment with rCTB had no significant effect on LPS-induced caspase-1 activation (0.42 ± 0.02 at OD_405_) or IL-1β secretion (73.3 ± 5.0 pg/ml) compared with those in cells treated with LPS + PBS (Figure 6). These results indicate that rCTB had no effect on caspase-1 activation or subsequent IL-1β secretion, and that the inhibitory effect of YVAD on capase-1 was not abolished by the fusion of YVAD to CTB.

The expression and secretion of YVAD by bacteria have been limited by its low molecular weight. Therefore, we constructed recombinant *L. casei* secreting YVAD as a fusion protein with CTB and showed that YVAD secreted by *L. casei* inhibits caspase-1 activation and subsequent IL-1β secretion. The results of this study indicate that YVAD secreted by bacteria exerts an anti-inflammatory effect. Meng *et al*. also reported that recombinant green fluorescent protein (GFP) fused to the C-terminus of CTB expressed in silkworms emitted green fluorescence similar to that emitted by recombinant GFP alone [18]. That report and the results of the present study suggest that the effects of functional peptides or proteins are not abolished by their fusion to the C-terminus of CTB.

## Conclusions

We constructed an *L. casei* that secretes a recombinant CTB protein fused to YVAD. Although rCTB–YVAD bound GM1 ganglioside and translocated into Caco-2 cells, like rCTB, rCTB–YVAD but not rCTB inhibited LPS-induced caspase-1 activation and subsequent IL-1β secretion without affecting cell viability. These results indicate not only that a recombinant fusion protein of CTB with a functional peptide secreted by *L. casei* has GM1-ganglioside-binding activity, but also that recombinant YVAD secreted by *L. casei* exerts an anti-inflammatory effect. The results of this study suggest that rCTB secreted by *L. casei* has potential utility as a system for the delivery of YVAD into IECs. However, we were unable to examine the anti-inflammatory effects of rCTB–YVAD-secreting *L. casei* in this study because *L. casei* could not grow or secrete rCTB–YVAD in MEM. We confirmed that it is difficult to completely mimic the intestinal environment in *in vitro* experiments. Therefore, further studies, such as an *in vivo* study of the ingestion of rCTB–YVAD secreting *L. casei*, are required to examine the effects of rCTB–YVAD-secreting *L. casei*. If successful, such a study would confirm that rCTB-secreting *L. casei* has potential utility as a delivery system for functional peptides into the intestine.

## Methods

### Bacterial strains and culture conditions

The strains used in this study were *L. casei* ATCC 27092 and *E. coli* DH5α. *L. casei* was grown at 37°C in MRS broth to produce the recombinant strain or at 30°C in MRS/K (MRS with 0.2 M potassium phosphate buffer) to produce the cell culture supernatant [5]. Erythromycin (5 μg/ml) was added to MRS or MRS/K to select the recombinant strain. *E. coli* was grown at 37°C in LB medium with or without ampicillin (100 μg/ml).

### Construction of the rCTB–YVAD secretion vector

PCR was performed using the plasmid pSCTB [5] as the template DNA, KOD-Plus- DNA polymerase (Toyobo, Osaka, Japan), and primers containing the YVAD-coding sequence fused to the C-terminus of the *CTB* gene: sense, 5′-caccaccaccaccaccactaaaggccttc-3′; anti-sense, 5′-atcagcaacataatttgccatactaattgc-3′. Initial denaturation was at 94°C for 2 min, followed by 30 cycles of denaturation at 94°C for 15 sec, annealing at 50°C for 30 sec, and extension at 68°C for 7 min. The DNA fragment of about 6,600 bp was separated by agarose gel electrophoresis and extracted from the gel with the QIAquick Gel Extraction Kit (Qiagen, Valencia, CA). The extracted DNA was phosphorylated at the 5′ end with T4 polynucleotide kinase (TaKaRa Bio, Shiga, Japan) and self-ligated with T4 DNA ligase (TaKaRa Bio). The rCTB–YVAD secretion vector pSCTB–YVAD was confirmed by sequencing, and then introduced into *L. casei* by electroporation, as described previously [5].

### Secretion of rCTB–YVAD by *L. casei*

Overnight cultures of *L. casei* transformed with pSCTB–YVAD or pSCTB were inoculated into MRS/K medium to an optical density at 600 nm (OD_600_) of 0.05, and the cells were grown at 30°C until they reached an OD_600_ of 2.0. The culture supernatants were collected by centrifugation (7,000 × *g*, 5 min, 4°C) and concentrated 10-fold with Amicon Ultra Centrifugal Filter Units (10 kDa; Merck Millipore, Tokyo, Japan). The secretion and specific GM1-ganglioside-binding activities of rCTB–YVAD and rCTB in the concentrated supernatant were confirmed with immunoblotting using an antibody directed against CT or a GM1 enzyme-linked immunosorbent assay (GM1-ELISA), respectively.

### Immunoblotting

The concentrated supernatants of *L. casei* (20 μl/lane), rCTB–YVAD (100 ng/lane), and cellular protein extracts of Caco-2 cells (50 μg/lane) were subjected to immunoblotting analysis. They were separated with SDS-PAGE (10–18%) and transferred to polyvinylidene difluoride membranes (GE Healthcare, Buckinghamshire, UK). Antibodies directed against CT (Sigma-Aldrich, St. Louis, MO) and β-actin (Cell Signaling Technology, Boston, MA) were used as the primary antibodies. Alkaline phosphatase (AP)-labeled anti-rabbit IgG antibody (Cell Signaling Technology) was used as the secondary antibody, and binding was detected with a chemiluminescent substrate of AP (CDP-Star Reagent; Biolabs, Beverly, MA).

### GM1-ELISA

A GM1-ELISA was performed to determine specific GM1-ganglioside-binding activities. Briefly, 96-well microtiter plates (Sumitomo Bakelite Co., Ltd., Tokyo, Japan) were coated with 5 μg/ml monosialoganglioside GM1 (Sigma-Aldrich) diluted in bicarbonate buffer (15 mM Na_2_CO_3_, 35 mM NaHCO_3_, pH 9.6), and incubated overnight at 4°C. After incubation, the plates were washed three times with PBS supplemented with 0.05% Tween-20 (PBS-T), and then blocked with PBS containing 1% bovine serum albumin (Nacalai Tesque, Kyoto, Japan) at 37°C for 2 h. After the plates were washed, the concentrated supernatant of *L. casei* (100 μl/well), rCTB–YVAD (50 ng/well), or rCTB (50 ng/well) was applied to the wells and incubated at 37°C for 2 h. The plates were incubated at 37°C for 2 h with antibody directed against CT and AP-labeled anti-rabbit IgG antibody used as the primary and secondary antibodies, respectively. The plates were incubated with AP substrate (Sigma-Aldrich) at 37°C for 20 min, and the OD_405_ was then measured with a microplate reader (ImmunoMini Nj-2300; Nunc, Rochester, NY).

### Purification of rCTB–YVAD secreted by *L. casei*

rCTB–YVAD from the culture supernatant of *L. casei* transformed with pSCTB–YVAD was purified using the His-tag and an affinity resin containing bound nickel ions. The culture supernatant of *L. casei* transformed with pSCTB–YVAD was collected by centrifugation (12,000 × *g*, 30 min, 4°C) after growth in MRS/K medium at 30°C until the OD_600_ was 2.0. After the supernatant was filtered at 0.22 μm, imidazole was added to a final concentration of 20 mM, and the culture supernatant was then adjusted to pH 7.0. Nickel resin (Ni Sepharose High Performance; GE Healthcare) was added to the culture supernatant and then mixed gently overnight at 4°C. The open column was filled with resin, and then washed with wash buffer (10 mM sodium phosphate, 500 mM NaCl, 20 mM imidazole, pH 7.4). rCTB–YVAD bound with nickel resin was eluted with elution buffer (10 mM sodium phosphate, 500 mM NaCl, 500 mM imidazole, pH 7.4). The eluted rCTB–YVAD was concentrated and the buffer replaced with PBS using Amicon Ultra Centrifugal Filter Units (10 kDa). The protein concentration of rCTB–YVAD was determined with Coomassie Protein Assay Reagent (Pierce, Perbio Science, Bonn, Germany). The purity of rCTB–YVAD was confirmed with SDS-PAGE on 17% polyacrylamide gel, followed by CBB staining and immunoblotting using an antibody directed against CT. The GM1-ganglioside-binding activities of purified rCTB–YVAD and rCTB were confirmed with GM1-ELISA.

### Caco-2 cell viability assay

Caco-2 cells were cultured as described previously [19]. Aliquots of 5 × 10^3^ Caco-2 cells were plated in each well of a 96-well plate (Nunc). The cells were treated with three concentrations of rCTB–YVAD or rCTB (10, 20, or 50 μM) in the presence of 10 μg/ml LPS from *E. coli* O55:B5 (Sigma-Aldrich). After incubation for 48 h, 20 μl of WST-1 Cell Proliferation Reagent (TaKaRa Bio) was added to each well. After 2 h, the OD_450_ and OD_630_ were measured with a microplate reader. Cell viability was calculated as (OD_450_ – OD_630_ of treated cells/OD_450_ – OD_630_ of untreated control cells) × 100%.

### Detection of translocated rCTB–YVAD and rCTB in Caco-2 cells

Aliquots of 7 × 10^5^ Caco-2 cells were plated in each well of six-well plates (Nunc). Cells were treated with 50 μM rCTB–YVAD or rCTB in the absence or presence of 10 μg/ml LPS. After incubation for 6 h, cellular protein extracts were prepared with PRO-PREP Protein Extraction Solution (iNtRON Biotechnology, Kyungki-Do, South Korea), according to the manufacturer’s protocol. The protein concentrations of the cellular protein extracts were determined with Coomassie Protein Assay Reagent. Intracellular rCTB–YVAD and rCTB were detected by immunoblotting with an antibody directed against CT. Equal loading was confirmed with an antibody directed against β-actin.

### Inhibitory effect on caspase-1 activity

Caspase-1 activity was determined with a modification of a previously described method [20,21]. Aliquots of 1 × 10^7^ Caco-2 cells were plated in 90 mm plastic culture dishes (Nunc) and treated with or without 10 μg/ml LPS for 12 h. The cells were washed with PBS and resuspended in buffer W (20 mM HEPES, 10 mM KCl, 1.5 mM MgCl_2_, 1 mM EGTA, 1 mM EDTA, pH 7.4) supplemented with 10 mM DTT and 1 mM phenylmethylsulfonyl fluoride. The cells were incubated at 4°C for 15 min and disrupted by 20 passages through a 23G needle. The lysates were then centrifuged at 12,000 × *g* for 5 min at 4°C and the supernatants collected. The protein concentrations of the lysates were determined with Coomassie Protein Assay Reagent. Aliquots of 10 μg/μl lysate were incubated at 30°C for 2 h in the absence or presence of 50 μM rCTB–YVAD or rCTB. The caspase-1 activity in the 10-fold-diluted lysate was determined with a Caspase 1 Assay Kit, Colorimetric (Calbiochem, La Jolla, CA).

### Measurement of IL-1β by ELISA

Aliquots of 7 × 10^5^ Caco-2 cells were plated in each well of six-well plates. The cells were treated with 50 μM rCTB–YVAD and rCTB in the presence of 10 μg/ml LPS for 48 h. The cell supernatants were centrifuged at 15,000 × *g* for 5 min at 4°C and stored at −80°C until IL-1β analysis. The concentrations of IL-1β in the cell supernatants were determined with a human IL-1β ELISA Kit (R&D Systems, Abingdon, UK).

### Statistical analyses

Data are presented as means  ±  SEM. Statistical analyses were performed with Origin Pro 8.1 (OriginLab, Northampton, MA). Differences were analyzed with one-way ANOVA followed by Tukey’s test. In all analyses, *P* < 0.05 was deemed to indicate significance.

## Abbreviations

BB: Serum albumin binding region; CBB: Coomassie Brilliant Blue; CT: Cholera toxin; CTA: Cholera toxin A subunit; CTB: Cholera toxin B subunit; GFP: Green fluorescent protein; GM1-ELISA: GM1 enzyme-linked immunosorbent assay; His-tag: Six-histidine-residue tag; IECs: Intestinal epithelial cells; IL-1β: Interleukin-1β; LAB: Lactic acid bacteria; LDH: Lactate dehydrogenase; LPS: Lipopolysaccharide; pro-caspase-1: Precursor of caspase-1; pro-IL-1β: Precursor of IL-1β; rCTB: Recombinant CTB; rCTB–YVAD: Recombinant fusion protein of CTB with YVAD; SS: Secretory signal sequence; Term: Terminator region; YVAD: Tetrapeptide composed of tyrosine, valine, alanine, and aspartic acid.

## Competing interests

The authors declare that they have no competing interests.

## Authors’ contributions

YH, MY, TS, and NK designed the research; YH, AK, SU, and YF acquired the data; YH analyzed the data; YH, TS, and KI drafted the manuscript. AT, TM, FM, and TA clarified the manuscript. All authors read and approved the final manuscript.

## Supplementary Material

Additional file 1: Figure S1 Culture of *L. casei* and its secretion of rCTB–YVAD or rCTB in MRS/K or MEM. The data confirm that *L. casei* does not grow or secrete rCTB–YVAD when cultured in MEM. Click here for file
