# Supplementary material for: Recombinant fusion protein of cholera toxin B subunit with YVAD secreted by Lactobacillus casei inhibits lipopolysaccharide-induced caspase-1 activation and subsequent IL-1 beta secretion in Caco-2 cells
Source: BMC Biotechnol. 2014 May 10;14:38. doi: 10.1186/1472-6750-14-38 (PMC4031163; doi:10.1186/1472-6750-14-38)
Supplement: Additional file 1: Figure S1 — Culture of L. casei and its secretion of rCTB–YVAD or rCTB in MRS/K or MEM. The data confirm that L. casei does not grow or secrete rCTB–YVAD when cultured in MEM. [file 1472-6750-14-38-S1.pdf]

A

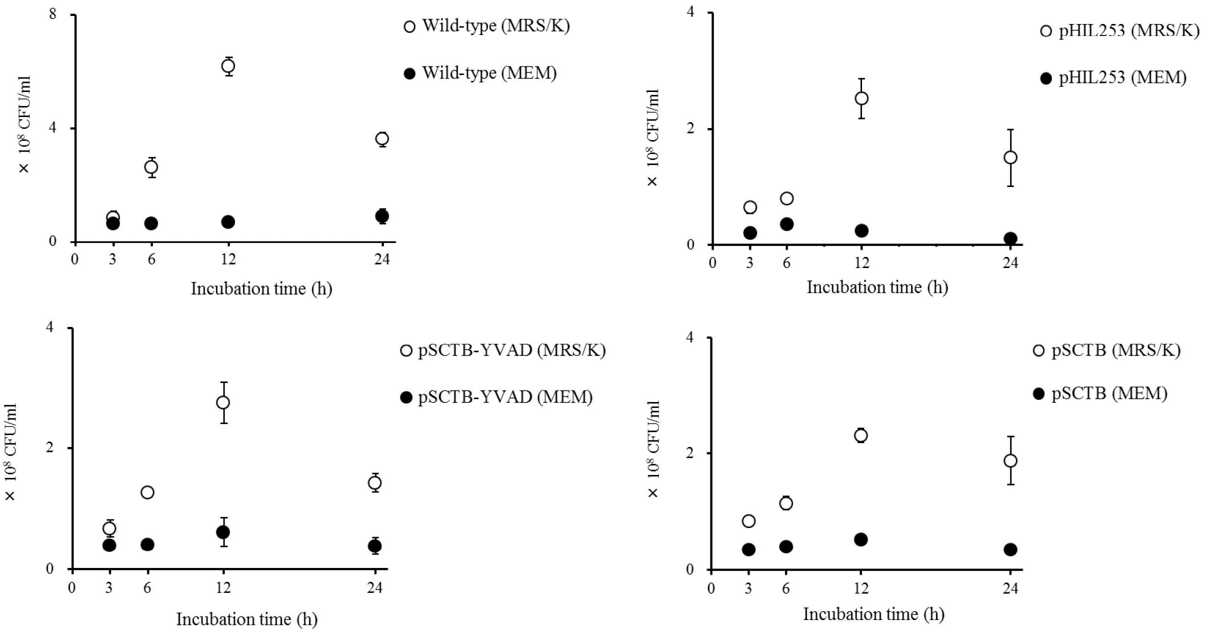

B

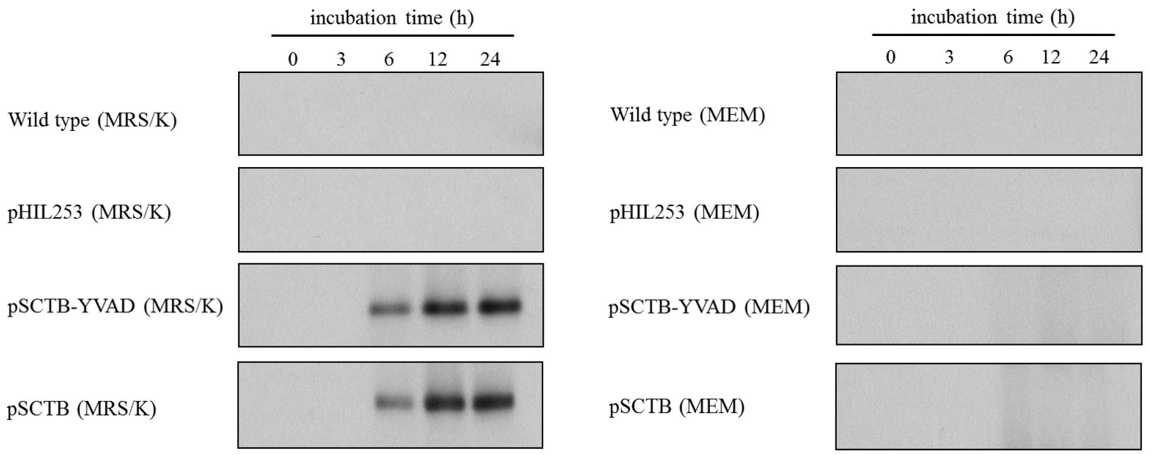

Supplementary figure 1 – Culture of *L. casei* and secretion of rCTB-YVAD and rCTB by *L. casei* in MRS/K or MEM.

Overnight cultures of wild-type *L. casei* and *L. casei* transformed with pHIL253, pSCTB-YVAD, and pSCTB were inoculated into MRS/K or MEM with or without erythromycin to OD_{600} of 0.05. Cells were grown at 37°C for 3, 6, 12, and 24 h, and plated onto MRS agar. After culture at 37°C for 2 days, the colonies were counted and the number of colony forming units (CFU) was calculated. Data represent the means ± SEM of two separate experiments performed in triplicate (A). The culture supernatants were collected and then concentrated tenfold. Western blotting using an antibody to CT was performed to analyze the secretion of rCTB-YVAD and rCTB in the supernatants. Data are representative of two separate experiments (B).
